# Covalent Organic Framework Bispecific Nanosystem for the Combined Treatment of Acute Myeloid Leukemia

**DOI:** 10.3390/ma19143001

**Published:** 2026-07-12

**Authors:** Huiyuan Bai, Mengsi Lin, Yiming Xia, Xi Gu, Maorong Jiang, Dengbing Yao

**Affiliations:** 1 School of Life Sciences, Key Laboratory of Neuroregeneration of Jiangsu and Ministry of Education, Co-Innovation Center of Neuroregeneration, Nantong University, Nantong 226019, China; ntdxlotus@163.com (X.G.); jiangmr@ntu.edu.cn (M.J.); 2 Medical School of Nantong University, Nantong University, Qixiu Campus, Nantong 226001, China; wsdmn18@126.com (M.L.); 18822355805@163.com (Y.X.)

**Keywords:** covalent organic framework, bispecific nanosystem, combined, acute myeloid leukemia

## Abstract

**Highlights:**

- The nanosystem possessed strong cascade catalytic activity to facilitate efficient chemodynamic therapy.
- The bone marrow stromal cell membrane component suppressed the migration and adhesion of C1498 cells by interfering with the CXCR4/CXCL12 axis.
- The nanosystem enhanced T cell-mediated cytotoxicity.

**Abstract:**

Drug resistance remains a significant challenge in the clinical treatment of acute myeloid leukemia (AML). Therefore, there is an urgent need to develop a novel combinatorial therapy strategy, aiming to overcome drug resistance and improve therapeutic outcomes in AML. Herein, we developed a covalent organic framework bispecific nanosystem, namely glucose oxidase-loaded iron porphyrin covalent organic framework coated with bone marrow stromal cell membrane and functionalized with anti-CD3 and anti-PD-L1 antibodies (abbreviated FeC-G@M-C&P). The fabricated FeC-G@M-C&P displayed good cascade catalytic activity. The bone marrow stromal cell membrane endowed the nanosystem with robust targeting ability, which further triggered abundant reactive oxygen species (ROS) production for chemodynamic therapy. Moreover, bone marrow stromal cell membrane component suppressed the migration and adhesion of C1498 cells by interfering with the CXCR4/CXCL12 axis. Meanwhile, anti-CD3 and anti-PD-L1 antibodies improved T cell activation, relieved immune suppression, and jointly enhanced T cell-mediated immune responses against leukemia cells. Experimental results indicated that the FeC-G@M-C&P plus T cells group showed better anti-leukemia effects compared with other groups, which can be attributed to the integration of chemodynamic therapy, CXCR4/CXCL12 axis blockade therapy and immunotherapy. Collectively, the fabricated nanosystem provided a promising approach for the combined treatment of AML.

## 1. Introduction

Acute myeloid leukemia (AML) is a fatal hematologic cancer characterized by clonal expansion and differentiation arrest of hematopoietic stem cells and progenitor cells [1,2]. To date, the main clinical treatment options include chemotherapy, targeted therapy and hematopoietic stem cells transplantation. Although the remission rate among treated patients has improved, the prognosis of elderly patients remains unsatisfactory. With the continuous advancements of anti-tumor technology, numerous novel treatment approaches, such as chemodynamic therapy (CDT), starvation therapy and immunotherapy, have been widely used to enhance tumor treatment outcomes [3,4]. Monotherapy is initially highly effective; however, resistance to monotherapy inevitably develops over time [5]. In contrast, therapies that integrate different mechanisms of action can inhibit and eliminate tumor cells through multiple pathways, holding promise for avoiding drug resistance and enhancing AML therapeutic efficacy. Furthermore, CXCR4/CXCL12 axis contributes to AML resistance. Most types of AML cells highly express CXCR4, while bone marrow stromal cells secrete its ligand, CXCL12 [6]. Owing to the interactions between CXCR4 and CXCL12, leukemia cells migrate and adhere to the bone marrow microenvironment, where leukemia cells subsequently obtain survival, proliferation and drug resistance signals [7,8]. Therefore, it is urgent to design a combination treatment strategy to combat drug resistance and enhance AML therapeutic efficacy.

In recent years, various blocking strategies against CXCR4/CXCL12 axis have been explored to inhibit tumor cell metastasis. For example, Gu et al. constructed breast cancer cell membrane-modified quantum dots to block CXCR4/CXCL12 axis for evaluating lung metastasis of breast cancer [9]. They found that the quantum dots inherited the CXCR4 expression of breast cancer cells, enabling them to effectively bind to CXCL12 protein and inhibit CXCL12-induced tumor cells metastasis. Motivated by this research, targeting and inhibiting CXCR4 by bone marrow stromal cell membrane holds promise for disturbing CXCR4/CXCL12 axis, which could block leukemia cells from returning to the protective bone marrow environment.

Immunotherapy is a powerful anti-tumor strategy that has achieved breakthrough in the treatment of hematologic malignancies. Among various cancer immunotherapies, bispecific antibodies represent a promising therapeutic approach. Bispecific antibodies are engineered antibodies that recognize two distinct targets. For example, bispecific T cell-redirecting antibodies, a subclass of bispecific antibodies, simultaneously target CD3 on T cells and tumor-specific antigen on tumor cells [10]. By bridging T cell and tumor cell, bispecific T cell-redirecting antibodies trigger T cell activation to dissociate tumor cell [11]. T cell-redirecting antibodies, such as Her2/CD3, CD123/CD3, and CD20/CD3, have been explored for tumor treatment [12,13,14]. However, their applications are faced with major challenges including complex production processes, high fabrication cost and rapid blood clearance. Chemodynamic therapy (CDT) is an emerging tumor therapy technology that converts hydrogen peroxide (H_2_O_2_) into poisonous hydroxyl radicals (·OH) via a Fenton or Fenton-like reaction, thereby expanding the oxidative stress reaction in tumor cells and specifically inducing tumor cell death [15,16]. Nanozymes, a class of nanomaterials with enzyme-like activity, have shown great therapeutic potential for CDT [17]. For example, peroxidase (POD)-like nanozyme can decompose H_2_O_2_ into highly toxic ·OH to induce tumor cell apoptosis, thus achieving CDT [18]. To date, POD-like nanozymes have made significant progress for tumor therapy because of their advantages including mild catalytic conditions, good stability and low fabrication cost. Nevertheless, both the limited H_2_O_2_ and acidity of the tumor microenvironment restrict the generation of adequate ·OH, leading to poor therapeutic effect [19]. It has been reported that glucose oxidase (GOX) catalyzes intracellular glucose to produce gluconic acid and H_2_O_2_, thereby providing sufficient substrate and acidity for the following POD-like catalytic reaction [20,21]. Hence, GOX can serve as a reinforced strategy for CDT.

Nanotechnology offers advantages in improving the pharmacokinetic behavior and stability of loaded therapeutic cargos [22,23]. Moreover, it provides a platform for combination therapy with multiple mechanisms of action [24]. On one hand, nanocarriers exert intrinsic POD-like activity for CDT. On the other hand, their structural characteristics facilitate loading of therapeutic cargos with different mechanisms. Porphyrins are ideal materials to construct high-efficiency nanozymes due to their advantages of porous structural features and outstanding catalytic performance and good biocompatibility. Porphyrins are conjugated macrocycles formed by four pyrrole rings through methylene bridges in which the four nitrogen atoms can form efficient and stable coordination with metal ions. Among these metal ions, the coordination compatibility of porphyrins with iron ions is particularly outstanding, which enables rapid interconversion between Fe^2+^ and Fe^3+^ and highly mimics the active center of natural enzymes. It has been demonstrated that iron porphyrins possess excellent redox catalytic properties and mimic multiple enzymes such as POD, catalase and monooxygenase [25]. Benefiting from these advantages of unique structure, outstanding catalytic ability and good biosafety, iron porphyrins show great potential for biomedical applications including antioxidant, antibacterial therapy and biosensing. Covalent organic frameworks are a new type of crystalline porous material assembled from organic units linked by strong covalent bonds, which were regarded as promising platforms due to their large surface areas, good structural stability, adjustable pore size and structural designability [26,27]. Encouraged by the unique features of porphyrins, porphyrins and their metallic derivatives can be integrated into covalent organic frameworks as organic ligands to endow them with good catalytic ability. So far, it has been confirmed that a covalent organic framework chelating iron with porphyrin exhibits enhanced catalytic activity [28,29].

**Scheme 1:**
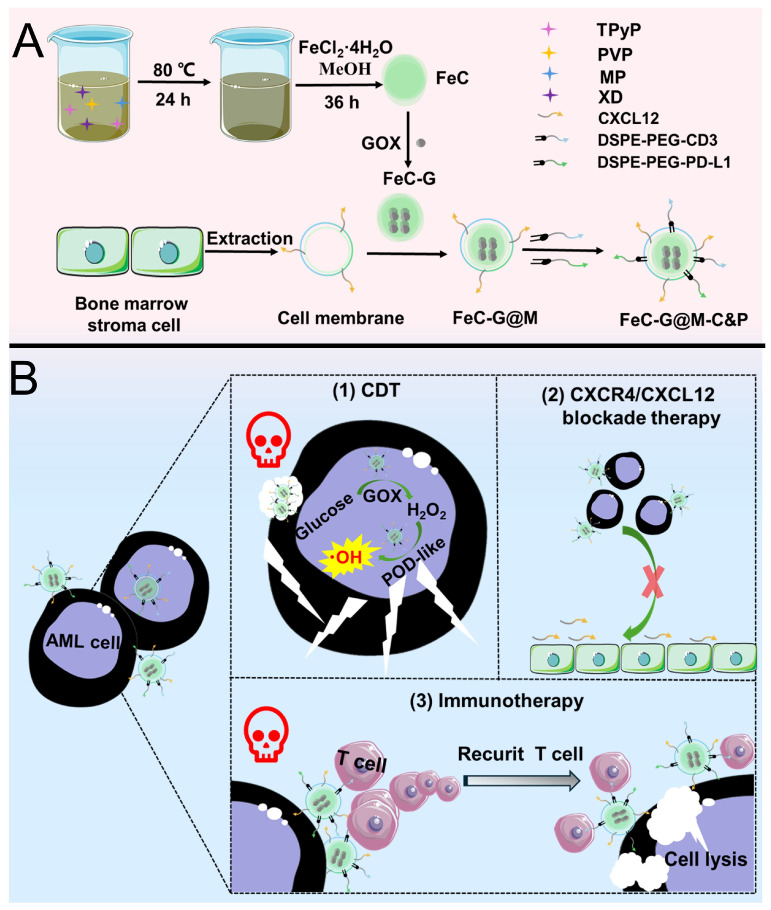
(**A**) Schematic of the preparation procedure of covalent organic framework bispecific nanosystem. (**B**) Schematic of the combined anti-leukemia application of covalent organic framework bispecific nanosystem.

Herein, we designed a covalent organic framework bispecific nanosystem for the combined treatment of AML (Scheme 1). The synthetic procedures were described as follows. First, iron porphyrin-based covalent organic framework (abbreviated FeC) was prepared by a facile method. Then GOX was encapsulated to prepare GOX-loaded FeC (abbreviated FeC-G). Subsequently, bone marrow stromal cell membrane was employed to prepare membrane coated FeC-G (abbreviated FeC-G@M). Finally, anti-CD3 and anti-PD-L1 antibodies pre-modified with 1,2-distearoyl-sn-glycero-3-phosphoethanolamine-(polyethylene glycol)-NHS ester (DSPE-PEG-NHS) were anchored into the phospholipid bilayer via physical interactions to prepare FeC-G@M modified with anti-CD3 and anti-PD-L1 antibodies (abbreviated FeC-G@M-C&P). The FeC core triggered the Fenton-like reaction in the acidic tumor microenvironment to produce ROS. Meanwhile, GOX depleted glucose and generated abundant H_2_O_2_ to amplify CDT induced by FeC. Bone marrow stromal cell membrane endowed the nanosystem with strong targeting ability, which aimed at enhancing CDT efficacy and realizing CXCR4/CXCL12 blockade therapy. Anti-CD3 and anti-PD-L1 antibody moieties simultaneously recognized CD3 on the surface of T cells and PD-L1 on the surface of leukemia cells to form intercellular crosslink between T cells and leukemia cells, narrowing cell–cell distance and activating anti-leukemia immune responses. The nanosystem achieved combined therapeutics effects of enhanced CDT, CXCR4/CXCL12 blockade therapy and immunotherapy in pursuit of solving poor efficiency of single therapy and drug resistance.

## 2. Materials and Methods

### 2.1. Chemical Reagents

5,10,15,20-tetra(4-pyridyl)-21H,23H-porphine (TPyP), 3,3′,5,5′-tetramethylbenzidine (TMB), 2,2′-azino-bis-(3-ethylbenzthiazoline-6-sulphonate) (ABTS), DSPE-PEG-NHS and methanol (MeOH) were provided by Shanghai Aladdin Biochemical Technology Co., Ltd. (Shanghai, China). 1-methyl-2-pyrrolidinone (MP) was provided by Beijing J&K Scientific Co., Ltd. (Beijing, China). p-xylylene dibromide (XD) was provided by Tianjin Heowns Biochemical Technology Co., Ltd. (Tianjin, China). Polyvinylpyrrolidone (PVP), ferrous chloride tetrahydrate (FeCl_2_·4H_2_O), and glucose were brought from Sinopharm Chemical Reagent Co., Ltd. (Shanghai, China). GOX and methyl red were brought from Shanghai Macklin Biochemical Co., Ltd. (Shanghai, China). Anti-CD3 and anti-PD-L1 antibody were brought from Bio X Cell (NH, USA). FITC-NHS was brought from Shanghai Toyang Biotechnology Co., Ltd. (Shanghai, China). Cy5-NHS was brought from Dalian Meilun Biotechnology Co., Ltd. (Dalian, China). Anti-CXCL12 antibody was brought from Beijing Biosynthesis Biotechnology Co., Ltd. (Beijing, China). Mouse leukemia cell (C1498) and human umbilical vein endothelial cells (HUVECs) were purchased from Shanghai Zhong Qiao Xin Zhou Biotechnology Co., Ltd. (Shanghai, China). Mouse Stromal-5 (MS-5) were provided by Shanghai Qingqi Biotechnology Development Co., Ltd. (Shanghai, China). Dulbecco’s modified eagle medium (DMEM) and Annexin V-FITC/PI apoptosis detection kit were purchased from Nanjing Keygen Biotech Co., Ltd. (Nanjing, China). Fetal bovine serum (FBS) was purchased from Hangzhou Si Ji Qing Biological Engineering Materials Co., Ltd. (Hangzhou, China). Penicillin-streptomycin solution was purchased from Wuhan Servicebio Technology Co., Ltd. (Wuhan, China). Iron assay kit was supplied by Nanjing Jiancheng Bioengineering Institute. Cell counting kit-8 (CCK-8), ROS assay kit and BCA protein assay kit were supplied by Shanghai Beyotime Biotechnology Co., Ltd. (Shanghai, China). CXCL12 was supplied by BD Bioscience (San Jose, CA, USA). Enzyme-linked immunosorbent assay (ELISA) kits for tumor necrosis factor-alpha (TNF-α), interleukin-2 (IL-2), and interferon-gamma (IFN-γ) were purchased from Abbkine Scientific Co., Ltd. (Wuhan, China). ELISA kits for perforin and granzyme B were purchased from Wuhan Huamei Biotech Co., Ltd. (Wuhan, China).

### 2.2. Isolation of Bone Marrow Stromal Cell Membrane

Cell membranes were isolated by hypotonic treatment and liquid nitrogen freeze–thaw method. MS-5 cells were collected by centrifugation, and thoroughly suspended in sterile 0.1 × PBS solution. Then cell suspensions were placed in a liquid nitrogen canister to freeze for 8 s, followed by fully thawing at room temperature. The process was repeated 6 times to destroy cell membrane. The obtained cell suspension was centrifuged at 12,000 rpm/min for 20 min at 4 °C. After discarding the supernatant, 1 mL of sterile water was added to suspend the cell membranes. BCA protein assay kit was used to measure protein concentration. The resulting cell membrane samples were kept at −80 °C for further use.

### 2.3. Preparation of FeC, FeC-G and FeC-G@M

30 mg of TPyP and 365 mg of PVP were dissolved in a flask containing 15 mL of MP. Then, 40 mg of XD was added into the flask, and stirred for 20 min. After purging the mixture with nitrogen gas for 15 min, the flask was placed in the water bath at 80 °C for 24 h. At the end of the reaction, the mixture was centrifuged, and the supernatant was discarded. Next, 40 mg of FeCl_2_·4H_2_O and 5 mL of MeOH were added into the obtained composite, and subsequently stirred at room temperature for 36 h. Upon completion of the reaction, the mixture was centrifuged, and the supernatant was discarded. The product FeC was collected after washing with MeOH. To prepare FeC-G, GOX and FeC were mixed at the mass ratio of 1:1, and stirred for 2.5 h at room temperature. FeC-G@M was prepared by incubating FeC-G with 0.5 mg of cell membranes at the mass ratio of 1:1 in an ultrasonic bath for 5 min.

For the preparation of FITC-labeled FeC, FITC and FeC were mixed at a mass ratio of 1:10 and stirred at room temperature in the dark overnight. Afterwards, the obtained product was centrifuged and thoroughly washed to remove free FITC. The preparation of FITC-labeled FeC@M was almost the same as that of FITC-labeled FeC, except that the product was further incubated with 0.5 mg of isolated cell membranes at a mass ratio of 1:1 in an ultrasonic bath for 5 min.

### 2.4. Preparation of FeC-G@M-P and FeC-G@M-C&P

First, antibodies were modified with DSPE-PEG-NHS via amide reaction. Specifically, DSPE-PEG-NHS was mixed with 100 μg of anti-CD3 antibody and 100 μg of anti-PD-L1 antibody with a molar ratio of 5:1 respectively, and the pH was adjusted to 8 by adding sodium bicarbonate solution. After incubation on a shaker at room temperature for 2 h, DSPE-PEG-NHS modified anti-CD3 antibody (DSPE-PEG-CD3) and anti-PD-L1 antibody (DSPE-PEG-PD-L1) were obtained via ultrafiltration. Then, the obtained antibodies were incubated with the resulting FeC-G@M at 37 °C for 1.5 h. The FeC-G@M-C&P was finally collected after centrifugation. At the same time, FeC-G@M modified with anti-PD-L1 antibody (abbreviated FeC-G@M-P) was prepared for comparison. The preparation procedure of FeC-G@M-P was almost the same as that of FeC-G@M-C&P except that DSPE-PEG-CD3 was replaced with DSPE-PEG-NHS, which anchored onto the phospholipid bilayer via physical interaction.

The preparation procedure of FeC@M and FeC@M-P were almost the same as that of FeC-G@M and FeC-G@M-P except the removing of GOX. The preparation of FeC-G@M modified with FITC-labeled anti-CD3 and Cy5-labeled anti-PD-L1 antibodies (abbreviated FeC-G@M-C_F_&P_C_) was almost the same as that of FeC-G@M-C&P except that FITC-NHS and DSPE-PEG-NHS co-modified anti-CD3 antibody as well as Cy5-NHS and DSPE-PEG-NHS co-modified anti-PD-L1 antibody were used.

### 2.5. Characterization

Transmission electron microscopy (TEM) images were captured by a JEM-1200EX electron microscope (Tokyo, Japan). X-ray photoelectron spectroscopy (XPS) measurements were detected by a Thermo Escalab 250Xi (, DE, USA). The polydispersity index (PDI) and zeta potential were measured by a Zetasizer Nano ZS90 (Malvern, UK). Thermal gravimetric analysis (TGA) was analyzed by a STA 449 F3 thermal analyzer (Selb, Germany). Fluorescence spectra were detected by an RF-5301PC luminescence spectrometer (Kyoto, Japan). Absorption spectra were obtained by a Nicolet evolution 300 spectrophotometer (Madison, WI, USA). CCK-8 experiment was conducted using an EnSpire 2300 microplate detector (Waltham, MA, USA). The expression of CXCL12 was detected by Western blotting using a ChemiDoc™ MP imaging system, Hercules, CA, USA).

### 2.6. Evaluation of POD-like Activity and Cascade Catalytic Activity

The following POD-like activity was evaluated by catalyzing ABTS/TMB oxidation method.

*Detection of POD-like activity of FeC:* For ABTS oxidation experiment, the catalytic system was configured as follows: the mixture of H_2_O_2_ (20 mM), FeC (500 μg/mL), ABTS (5 mg/mL), and buffer at a volume ratio of 1:1:1:7 was prepared, followed by incubating at room temperature for 10 min. Set groups without the addition of H_2_O_2_ or FeC as control. Ending incubation, absorption spectra of the solution were recorded by ultraviolet–visible spectrophotometer. For the TMB oxidation experiment, the operation was the same as that of ABTS excepting the replacement of ABTS by TMB.

*Effect of H_2_O_2_ concentrations on POD-like activity of FeC*: For ABTS oxidation experiment, the catalytic system was configured as follows: the mixture of H_2_O_2_ with various concentrations, FeC (250 μg/mL), ABTS (5 mg/mL), and buffer at the volume ratio of 1:1:1:7 was prepared, followed by incubating at room temperature for 25 min. Ending incubation, absorption spectra of the solution were recorded by ultraviolet–visible spectrophotometer. For the TMB oxidation experiment, the operation was the same as that of ABTS excepting the replacement of ABTS by TMB.

*Effect of pH on POD-like activity*: The mixture of H_2_O_2_ (20 mM), FeC (250 μg/mL), TMB (5 mg/mL), and buffers of different pH at a volume ratio of 1:1:1:7 was prepared, followed by incubating at room temperature for 10 min. Ending incubation, absorbance at 652 nm of the solution was recorded by a microplate reader.

*Acidity changes in the reaction system catalyzed by FeC-G@M-C&P:* The catalytic system was configured as follows: the mixture of glucose (300 mM), FeC-G@M-C&P (500 μg/mL), and buffer at a volume ratio of 1:2:7 was prepared, followed by incubating at room temperature for 24 h. Set the group without the addition of glucose as control. Ending incubation, methyl red indicator was added to the reaction solution. The color was photographed and recorded.

*Cascade catalytic activity of FeC-G@M-C&P:* The experimental group was prepared by mixing glucose (300 mM), FeC-G@M-C&P (500 μg/mL), TMB (5 mg/mL), and buffer at a volume ratio of 1:1:1:7. Glucose + TMB, FeC-G@M-C&P + TMB, and Glucose + GOX + TMB groups were set as controls. The mixture was incubated at room temperature for 10 min, and the absorbance at 652 nm of the solution was recorded by a microplate reader.

*Effect of temperature on POD-like activity*: Preparation of the catalytic system was the same as that in the upper part. After incubation at 4 °C or 37 °C for 10 min, the absorbance at 652 nm of the solution was recorded by a microplate reader.

### 2.7. Cell Culture

C1498 cells were incubated in DMEM supplemented with 1% streptomycin–penicillin solution and 10% FBS. MS-5 cells and HUVECs were incubated in RPMI 1640 medium supplemented with 1% streptomycin–penicillin solution and 10% FBS. Cells were incubated in a humidified atmosphere containing 5% CO_2_ at 37 °C.

### 2.8. Evaluation of Targeting, ROS Generation Ability and Cell Apoptosis Rate

C1498 cells were seeded into 6-well plates at a density of 1 × 10^6^ cells/well. The cells of experimental groups were separately treated with FeC and FeC@M at a concentration of 100 μg/mL. PBS treated cells were served as control. After 4 h of treatment, cell suspensions were centrifuged at 1000 rpm/min for 5 min. The cells were collected and washed three times with sterile PBS. The content of iron was measured according to the instruction of the test kit, and the relative iron content was calculated.

C1498 cells were seeded into 6-well plates at a density of 1 × 10^6^ cells/well. The cells of experimental groups were separately treated with FITC-labeled FeC, FITC-labeled FeC@M and CXCL12 + FITC-labeled FeC@M at a concentration of 100 μg/mL. The concentration of CXCL12 was 5 μg/mL, with a 0.5 h pre-incubation prior to treatment. After 4 h of treatment, the cell suspensions were centrifuged at 1000 rpm/min for 5 min. The collected cells were washed three times with sterile PBS and detected by a Beckman Coulter CytoFLEX flow cytometer.

C1498 cells were seeded into 6-well plates at a density of 1 × 10^6^ cells/well. The cells of experimental groups were separately treated with Glucose + FeC-G and Glucose + FeC-G@M. The concentration of glucose and nanomaterial was 30 mM and 100 μg/mL, respectively. PBS treated cells were served as control. Twelve hours later, old culture media were replaced by that containing 10 μM DCFH-DA, and further incubated in the cell culture incubator. After 20 min of incubation, the cell suspensions were centrifuged at 1000 rpm/min for 5 min. The cells were washed three times. Fluorescence intensity was measured by a microplate (excitation: 488 nm; emission: 525 nm). The relative fluorescence intensity of cells was calculated.

C1498 cells were seeded in 6-well plates at a density of 1 × 10^6^ cells/well. The cells of experimental groups were separately treated with Glucose + FeC-G and Glucose + FeC-G@M for 24 h at a concentration of 100 μg/mL. The concentration of glucose was 30 mM. After treatment, cell suspensions were centrifuged at 1000 rpm/min for 5 min. The cells were collected and washed, and then incubated with 5 μL Annexin V-FITC and 5 μL PI at room temperature in the dark for 10 min. Cell apoptosis was determined using a Beckman Coulter CytoFLEX flow cytometer.

### 2.9. Evaluation of Migration and Adhesion Ability

For the migration assay, C1498 cells were seeded in 6-well plates. The cells of the experimental group were separately treated with FeC and FeC@M at a concentration of 100 μg/mL, while that of the control and blank group received no treatment. After 1 h of treatment, 3 × 10^5^ cells were inoculated into the upper chambers of the Transwell. The lower chambers of the experimental and control group were added with cell culture medium containing CXCL12 (0.2 μg/mL), while that of the blank group was added with an equal volume of blank cell culture medium. After 24 h of incubation, cells in the lower chambers were collected. CCK-8 reagent was added and placed the plate in the cell culture incubator in the dark for 3 h. The absorbance at 450 nm was measured by a microplate reader. Relative migration rate of cells was calculated.

For adhesion assay, MS-5 cells were seeded in 6-well plates. After formed stromal cell layers, 1 × 10^5^ cells were further seeded into cell culture plates. C1498 cells in the experimental group were pre-treated with FeC and FeC@M (100 μg/mL) for 1 h, respectively, while that in the control group received no treatment. After 24 h of incubation, non-adherent cells were gently removed. CCK-8 reagent was added and placed the plate in the cell culture incubator in the dark for 3 h. The absorbance at 450 nm was measured using a microplate reader. After deducting the absorbance of the group only containing MS-5 cells, relative adhesion rate of cells was calculated.

### 2.10. Evaluation of the Enhanced T Cell Cytotoxicity and Combined Anti-Leukemia Effect of FeC-G@M-C&P

C1498 cells (target cells) were seeded in 96-well culture plates at a density of 5 × 10^4^ cells/well. C1498 cells were co-incubated with different concentrations of FeC-G@M-C&P and T cells (effector cells). T cells were obtained from the spleens of healthy mice and expanded for two weeks prior to use. The effector/target ratio was set as 5:1. After 48 h of incubation, CCK-8 reagent was added into each well and placed the plate in the cell culture incubator for 3 h. Absorbance at 450 nm was measured using a microplate reader, and the cell viability was calculated.

C1498 cells were seeded in 96-well culture plates at a density of 5 × 10^4^ cells/well. To demonstrate the benefits of dual antibody loading, C1498 cells were randomly divided into the T cells group, FeC-G@M-P + T cells group and FeC-G@M-C&P + T cells group. The effector/target ratio was set as 5:1, and the concentration of the FeC-G@M-C&P was 100 μg/mL. After 24 h incubation, CCK-8 reagent was added into each well and placed the plate in the cell culture incubator for 3 h. Absorbance at 450 nm was measured using a microplate reader, and the cell viability was calculated.

C1498 cells were seeded in 96-well plates at a density of 5 × 10^4^ cells/well. The cells were randomly divided into the control, T cells, and FeC-G@M-C&P + T cells groups, and received corresponding treatments. The effector/target ratio was set as 5:1, and the concentration of the FeC-G@M-C&P was 100 μg/mL. After 48 h treatment, cell suspensions were centrifuged for 5 min at 4 °C. The supernatant was carefully collected, and the contents of TNF-α, IL-2, and IFN-γ in samples were determined following the manufacturer’s instructions of the ELISA kits. The levels of perforin and granzyme B in cell culture supernatants were measured by the identical procedure. The relative level of cytokines was calculated.

C1498 cells were seeded in 96-well plates at a density of 5 × 10^4^ cells/well. The cells were divided into the control group, Glucose + FeC-G, Glucose + FeC-G@M, Glucose + FeC-G@M-P, and the Glucose + FeC-G@M-C&P + T cells group. The effector/target ratio was set as 5:1. The concentration of glucose and nanomaterial is 30 mM and 25 μg/mL, respectively. After 24 h treatment, CCK-8 reagent was added and placed the plate in the culture incubator for 3 h. Absorbance at 450 nm was measured using a microplate reader, and the cell viability was calculated.

### 2.11. Evaluation of Biosafety and Biocompatibility

HUVECs were seeded in 96-well plates at a density of 5 × 10^4^ cells/well and cultured overnight. Subsequently, the old culture medium was replaced with a fresh medium containing different concentrations of FeC-G@M-C&P ranging from 0 to 100 μg/mL, while PBS-treated cells were set as the control group. After 48 h incubation, CCK-8 reagent was added to each well, and the plates were further incubated at 37 °C for 3 h in the dark. Finally, the absorbances at 450 nm were detected by a microplate reader to calculate the cell viability.

Fresh mouse blood was collected and centrifuged to harvest the red blood cells (RBCs), which were repeatedly washed with PBS to remove impurities. Then 2% RBCs was prepared for subsequent experiments. In detail, 500 μL RBCs was separately mixed with an equal volume of pure water, PBS or FeC-G@M-C&P dispersions with different concentrations. The pure water group was served as the positive control, and the PBS was served as the negative control. After incubation at 37 °C for 30 min, all mixtures were centrifuged at 3500 rpm for 5 min. The supernatants were transferred to a 96-well plate for visual observation, followed by measuring the absorbances at 545 nm by a microplate reader to calculate the hemolysis rate.

FeC-G@M-C&P was dispersed in PBS solution, followed by incubation at 37 °C. At predetermined time points (0, 12, 24, 48 h), 1 mL of sample dispersion was gently taken off and loaded into a disposable dynamic light scattering cuvette. The PDI was determined by Zetasizer Nano ZS90 (Malvern, UK).

### 2.12. Statistical Analysis

Data fitting and analysis were performed by GraphPad Prism 6 and SPSS Statistics 27 software. Data were presented as mean ± standard deviation. Briefly, one-way analysis of variance coupled with Tukey’s post hoc test was applied for statistical evaluation. * *p* < 0.05 and ** *p* < 0.01 represent that the difference was statistically significant.

## 3. Results and Discussions

### 3.1. Preparation and Characterization of FeC-G@M-C&P

**Figure 1:**
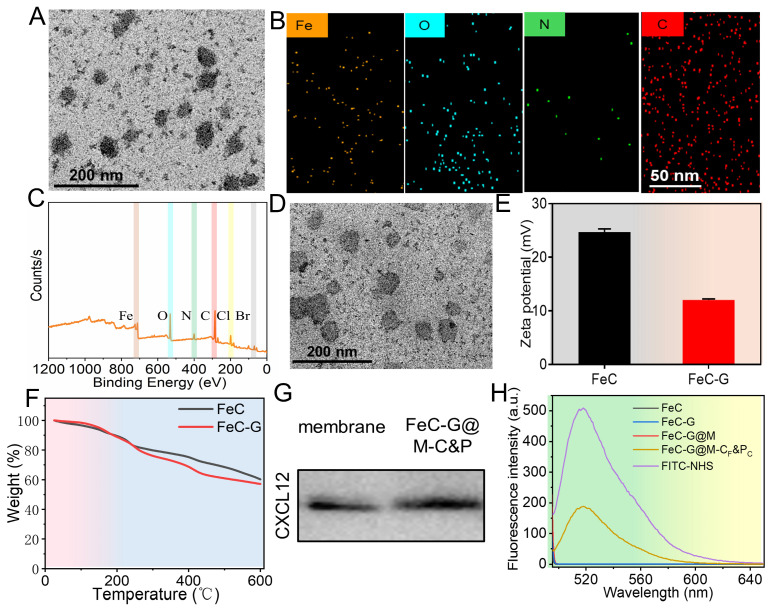
(**A**) TEM image of FeC. (**B**) EDS images of FeC. (**C**) XPS analysis of FeC. (**D**) TEM image of FeC-G. (**E**) Zeta potential values of FeC and FeC-G. (**F**) TGA curves of FeC and FeC-G. (**G**) Expression of CXCL12 protein measured by Western blotting. (**H**) Fluorescence spectroscopy of nanosystems with different formulations (excitation wavelength: 492 nm).

The synthesized procedure of FeC is illustrated in Scheme 1. FeC was synthesized by utilizing TPyP, PVP, MP, XD, FeCl_2_·4H_2_O, and MeOH via a facile method. Transmission electron microscopy (TEM) was performed to characterize the synthesized FeC. As shown in Figure 1A, FeC showed irregular shape with particle diameter less than 100 nm. The energy dispersive spectroscopic (EDS) image exhibited the constituent elements including Fe, O, N, and C, indicating the successful preparation of FeC (Figure 1B). X-ray photoelectron spectroscopy (XPS) was further employed to confirm the valence states and the constituent elements. Both the characteristic peaks of full and narrow spectra implied the existence of the mentioned constituent elements (Figure 1C). Moreover, we performed peak fitting on XPS spectra of Fe 2p. The results indicated the coexistence of Fe^2+^ and Fe^3+^ species (Figure S1). The ratio of Fe^2+^ was ~57%. The presence of Fe^2+^ provided critical active sites for the POD-like catalytic reaction [30,31].

Subsequently, GOX loaded FeC was obtained by mixing GOX with FeC under stirring condition at room temperature. It could be seen that GOX did not affect the size in nanoscale and dispersity (Figure 1D). Moreover, the polymer dispersity index (PDI) value of FeC-G was below 0.2, demonstrating its good dispersity (Figure S2). FeC and FeC-G displayed similar UV–vis absorption spectra, suggesting the negligible effects of GOX on the structure of FeC (Figure S3). The zeta potential decreased from 24.7 ± 0.6 to 12.0 ± 0.2 mV after adsorption GOX, which could be attributed to the negative charge of GOX biomolecular (Figure 1E and Figure S4). As shown in Figure 1F, the weight loss of FeC-G was greater than that of FeC above 200 °C, which was primarily ascribed to the decomposition of GOX.

It is essential to investigate whether FeC-G@M-C&P preserves CXCL12 protein of bone marrow stromal cell membrane. Then, we analyzed CXCL12 expression by Western blotting included the following important steps: protein extraction and concentration measurement, protein denaturation, sample loading, gel electrophoresis, membrane transfer, blocking, antibodies incubation and protein detection. As shown in Figure 1G, the protein band of the FeC-G@M-C&P group was basically consistent with that of cell membrane group. These phenomena indicated that CXCL12 protein of FeC-G@M-C&P can be well retained, which guaranteed the achievement of its biological function. To verify the successful functionalization of anti-CD3 and anti-PD-L1 antibodies on the surface of the nanosystem, FITC-labeled anti-CD3 antibody and Cy5-labeled anti-PD-L1 antibody were employed to construct FeC-G@M-C_F_&P_C_. As shown in Figure 1H and Figure S5, FeC-G@M-C_F_&P_C_ exhibited the similar emission spectra of the fluorescent molecules of FITC and Cy5 in the resulting spectra, indicating the successful conjugation of the two antibodies.

### 3.2. Evaluation of Cascade Catalytic Reaction

**Figure 2:**
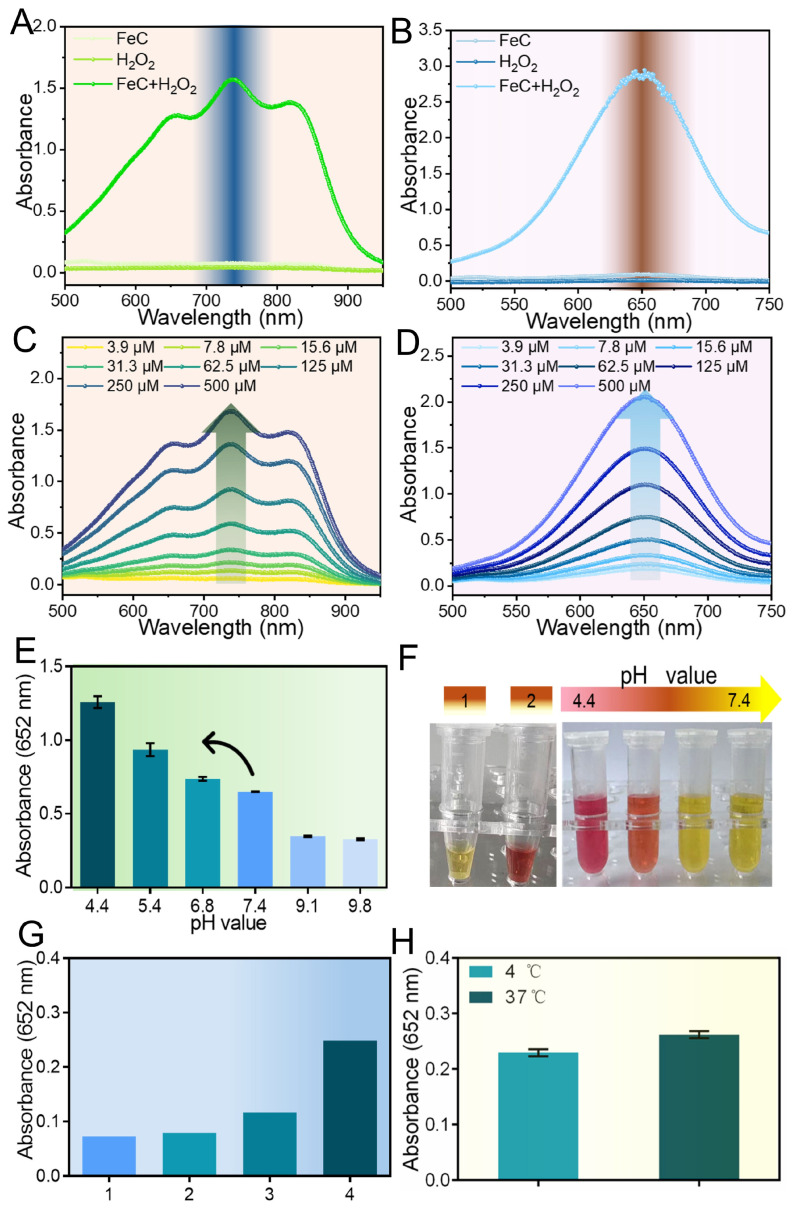
(**A**) Absorption spectra of ABTS solution mixed with FeC, H_2_O_2_, and FeC + H_2_O_2_, respectively. (**B**) Absorption spectra of TMB solution mixed with FeC, H_2_O_2_, and FeC + H_2_O_2_, respectively. (**C**) Absorption spectra of ABTS solution mixed with FeC and H_2_O_2_ with different concentrations. (**D**) Absorption spectra of TMB solution mixed with FeC and H_2_O_2_ with different concentrations. (**E**) Absorption of TMB solution mixed with FeC and H_2_O_2_ in buffers with different pH. (**F**) Images of the solutions incubated with methyl red. 1: FeC-G@M-C&P; 2: Glucose + FeC-G@M-C&P. From left to right of the below part are the pictures of the solutions with pH of 4.4, 5.4, 6.8, and 7.4, respectively. (**G**) From left to right is the absorbance of the TMB solution mixed with Glucose, FeC-G@M-C&P, Glucose + GOX and Glucose + FeC-G@M-C&P, respectively. (**H**) Absorbance of TMB solution mixed with glucose and FeC-G@M-C&P after incubation at 4 or 37 °C.

The cascade catalytic reaction induced by FeC-G@M-C&P is assumed as follows: GOX loaded in FeC-G@M-C&P catalyzed glucose and oxygen into gluconic acid and H_2_O_2_. Next, FeC in FeC-G@M-C&P utilized its POD-like activity to transfer the produced H_2_O_2_ into active ·OH. In this work, POD-like activity of FeC was firstly assessed by ABTS/TMB oxidation method. It is known that ·OH oxidized colorless ABTS/TMB into the colored oxidized ABTS/TMB product (oxABTS/oxTMB), which displayed the characteristic absorption at ~734 nm and ~652 nm separately. Therefore, POD-like catalytic performance can be reflected by detecting the characteristic absorption intensity. As shown in Figure 2A,B, almost no characteristic absorptions were observed in both FeC and H_2_O_2_ group, indicating FeC and H_2_O_2_ alone hardly catalyzed ABTS/TMB to oxidized form. Interestingly, there are obvious characteristic absorptions in FeC + H_2_O_2_ group, suggesting the formation of oxABTS/oxTMB. These results proved that FeC possesses POD-like activity.

We evaluated the effect of H_2_O_2_ concentrations on POD-like activity. In the case of ABTS oxidation, the characteristic absorption intensity continuously raised as the concentration of H_2_O_2_ increased (Figure 2C). The results in TMB oxidation experiment also displayed the characteristic absorption intensity enhanced with the increasing of H_2_O_2_ concentration (Figure 2D). In addition, we studied the effect of pH on POD-like activity. As shown in Figure 2E, the absorbance of oxTMB increased as the pH of the reaction solution decreased at the same reaction time, indicating that FeC exhibited stronger POD-like activity under acidic condition than that under neutral or alkaline conditions. This property is beneficial for antitumor application because the tumor microenvironment is acidic. Moreover, the relatively low POD-like activity in neutral environment is also helpful to prevent damage to normal cells.

Acidity changes in the reaction system catalyzed by FeC-G@M-C&P were evaluated by methyl red indicator. The methyl red indicator displayed red color below pH 4.4 and yellow color over pH 6.2. It is found that FeC-G@M-C&P enhanced the acidity of the reaction system containing glucose (Figure 2F). The results suggest that FeC-G@M-C&P efficiently catalyzed the conversion of glucose into gluconic acid, which was favorable for obtaining better catalytic activity. TMB oxidation method was used to test the glucose-activated cascade catalytic performance of FeC-G@M-C&P. The result showed that the introduction of glucose into the mixture containing FeC-G@M-C&P and TMB triggered cascade catalytic behavior of FeC-G@M-C&P as evidenced by obvious absorbance identified at 652 nm (Figure 2G). Temperature effects the catalytic performance of natural enzymes. Inspired by this, we evaluated the effect of temperature on the catalytic activity of FeC-G@M-C&P. As shown in Figure 2H, the absorbance of the TMB solution mixed with FeC-G@M-C&P and glucose increased as the temperature elevated from 4 to 37 °C, which provides the feasibility for the future application of in vivo study.

### 3.3. Assessment of Targeting Ability and ROS Generation Ability

**Figure 3:**
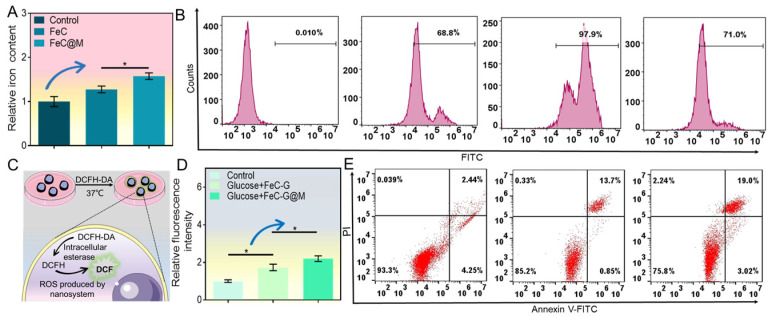
(**A**) Relative iron content of C1498 cells after treatment with FeC and FeC@M and for 4 h (*n* = 3). (**B**) Fluorescence intensity of C1498 cells after treatment with different nanoparticles for 4 h. The figures from left to right are blank, FITC-labeled FeC, FITC-labeled FeC@M, and CXCL12 + FITC-labeled FeC@M groups. (**C**) The detection principle of ROS produced by bispecific nanosystem. (**D**) Relative fluorescence intensity of C1498 cells after treatment with Glucose + FeC-G and Glucose + FeC-G@M for 12 h (*n* = 3). (**E**) Cell apoptosis of C1498 cells after treatment with different nanosystems for 24 h. The figures from left to right are control, Glucose + FeC-G and Glucose + FeC-G@M groups. * *p* < 0.05; ** *p* < 0.01.

Efficient uptake of therapeutic drugs by tumor cells is necessary for achieving superior therapeutic effect. Then, we assessed AML cells targeting capability and ROS generation ability. As illustrated in Figure 3A, the relative iron content of FeC@M group was significantly increased in comparison to the FeC group, indicating the enhanced cellular uptake of the latter. This phenomenon can be attributed to the targeting capability of bone marrow stromal cell membrane. Subsequently, flow cytometry analysis was conducted to verify CXCR4 receptor-mediated targeting. The results exhibited that the cellular fluorescence intensity of FITC-labeled FeC@M group was markedly higher than that of the FITC-labeled FeC group, confirming the strong specific recognition of FeC@M by C1498 cells. Notably, when cells were pre-incubated with free CXCL12 ligand to block surface CXCR4 receptors, the cellular fluorescence intensity of the CXCL12 + FITC-labeled FeC@M group was attenuated compared with the FITC-labeled FeC@M group, which was nearly comparable to that of the FITC-labeled FeC group (Figure 3B). These results revealed that the targeted accumulation of FeC@M was mediated by the CXCR4/CXCL12 receptor–ligand axis. Based on these results, we inferred that the improved targeting ability endowed by bone marrow stromal cell membrane is conducive to enhancing CDT therapeutic outcome of the therapeutics drugs.

To examine this possibility, ROS production was detected by using 2′,7′-dichlorodihydrofluorescein diacetate (DCFH-DA) probe, which was a classic probe to measure intracellular ROS. In detail, DCFH-DA is a cell-permeable agent, which can be hydrolyzed by cellular esterase to form DCFH and further oxidized by ROS to form fluorescent product (Figure 3C). Thus, the fluorescence intensity of the product is correlated with ROS level. We found fluorescence intensity in the Glucose + FeC@M group was stronger than other groups, suggesting enhanced ROS accumulation of the Glucose + FeC@M group (Figure 3D). These results indicated that the bone marrow stromal cell membrane modification effectively enhanced the internalization of therapeutics drugs, and thereby increased ROS production of tumor cells.

Annexin V/PI double staining is a classic and authoritative assay for evaluating ROS-triggered cell apoptosis. To confirm the apoptosis triggered by CDT-derived ROS, Annexin V/PI flow cytometric analysis was conducted. As shown in Figure 3E, the apoptotic cell proportion in the Glucose + FeC-G and Glucose + FeC-G@M groups reached 14.55% and 22.02%, respectively, while the apoptotic proportion of the control group was only 6.69%. The elevation of apoptotic cell proportion in Glucose + FeC-G and Glucose + FeC-G@M groups directly validated that ROS overproduction by the POD-like nanozyme initiated cell apoptosis.

### 3.4. Evaluation of Cell Migration and Adhesion Ability

**Figure 4:**
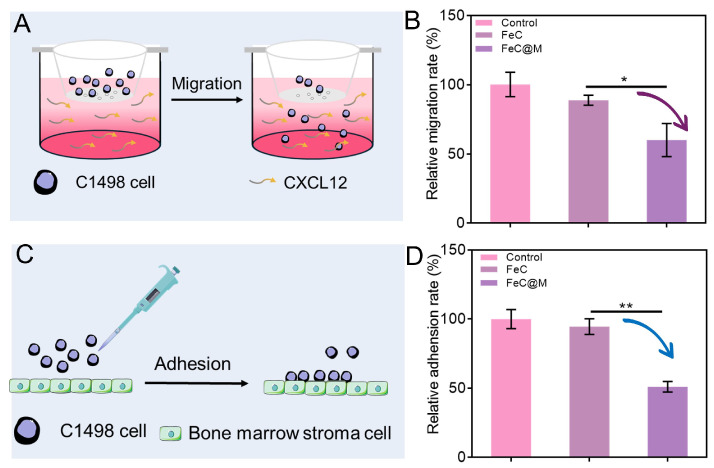
(**A**) A schematic diagram illustrating the migration of C1498 cells induced by CXCL12. (**B**) Relative migration rate of C1498 cells in different treatment groups (*n* = 3). (**C**) A schematic diagram illustrating the adhesion of C1498 cells induced by bone marrow stromal cell (**D**) Relative adhesion rate of C1498 cells to stromal cells in different treatment groups (*n* = 3). * *p* < 0.05; ** *p* < 0.01.

It is known that leukemia cells migrated and adhered to bone marrow microenvironment via CXCR4/CXCL12 axis to acquire proliferation and drug resistance signals. Herein, we firstly evaluated the effects of FeC@M on the migration of C1498 cells. A Transwell assay was conducted to evaluate cell migration behavior (Figure 4A). As shown in Figure 4B, the relative migration rate of the FeC group showed no significant change compared with the control, while relative migration rate of the FeC@M group was lower than that of the FeC group (60.1% ± 12.0% vs. 88.8% ± 3.6%). The result confirmed the inhibiting effect induced by cell membrane component on leukemia cells migration. Next, we evaluated the effects of FeC@M on the adhesion of C1498 cells. The MS-5 cells layer was prepared to mimic bone marrow as illustrated in Figure 4C. The relative adhesion rate of the FeC group showed a negligible decrease compared with the control group, while relative adhesion rate of the FeC@M group was significantly decreased in comparison to the FeC group (Figure 4D), indicating the blocking effect induced by the cell membrane component on leukemia cells adhesion. Overall, these results demonstrate that the chemotactic migration and adhesion of C1498 cells was obviously suppressed after FeC@M blocking, which strongly validated the dependence of cell recruitment on the CXCR4/CXCL12 axis.

### 3.5. Evaluation of T Cell-Mediated Cytotoxicity and Anti-Leukemia Effect

**Figure 5:**
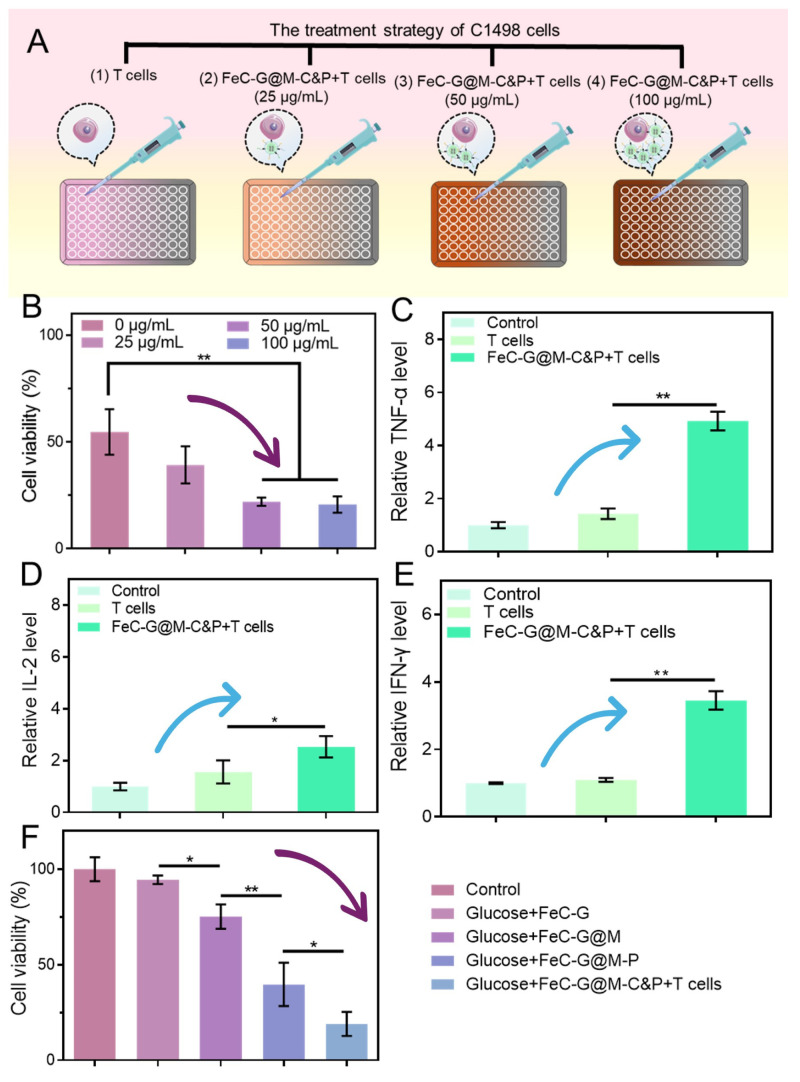
(**A**) Illustration of the treatment strategy of C1498 cells. (**B**) Viability of C1498 cells after incubation with different concentrations of FeC-G@M-C&P and T cells for 48 h (*n* = 3). Relative levels of (**C**) TNF-α, (**D**) IL-2 and (**E**) IFN-γ in cell culture supernatants in different treatment groups (*n* = 3). (**F**) Viability of C1498 cells in different treatment groups (*n* = 3). * *p* < 0.05; ** *p* < 0.01.

The mechanism of T cell activation by FeC-G@M-C&P relies on the co-localization of anti-CD3 and anti-PD-L1 antibodies. Anti-CD3 antibody binds to CD3 on the T cell surface to trigger T cell receptor signaling for T cell priming, and anti-PD-L1 antibody simultaneously blocks PD-L1/PD-1 axis for removing immune suppression, jointly maintains stable T cell-C1498 immunological synapses, and enhances anti-leukemic cytotoxicity. To facilitate in vitro T cell activation, T cells and C1498 cells were co-incubated at a fixed effector/target ratio to obtain dual-signal stimulation. We studied the effect of FeC-G@M-C&P on T cell activation and T cell-mediated killing. The treatment strategy is shown in Figure 5A. After treatment, CCK-8 reagent was added to evaluate the cell viability. As shown in Figure 5B, the viability of C1498 cell gradually reduced with the increasing concentrations of FeC-G@M-C&P. Notably, the 50 μg/mL and 100 μg/mL groups exhibited a significant decrease in cell viability relative to the control group (0 μg/mL). These data confirmed that FeC-G@M-C&P can enhance T cell-mediated killing in a dose-dependent manner.

Next, we established three groups (T cells, FeC-G@M-P + T cells, FeC-G@M-C&P + T cells) to verify the superiority of dual-antibody loading. It was observed that the cell viability exhibited a clear descending trend: T cells > FeC-G@M-P + T cells > FeC-G@M-C&P + T cells (Figure S6). Cell viability of the FeC-G@M-P + T cells group was lower than that of the T cells group, which was attributed to the alleviation of PD-L1-mediated immune suppression. Notably, cell viability was markedly reduced in the FeC-G@M-C&P + T cells group versus the FeC-G@M-P + T cells group, indicating the therapeutic advantages of FeC-G@M-C&P + T cells group. The results demonstrated that FeC-G@M-C&P simultaneously delivered anti-CD3 antibody for T cell activation and anti-PD-L1 antibody for checkpoint blockade to enhance anti-leukemic effect.

The superiority of FeC-G@M-C&P benefits from the contribution of anti-CD3 and anti-PD-L1 antibodies to immunological synapses formation. FeC-G@M-C&P exerts prominent regulatory effects on the formation of T cell-C1498 immunological synapses. Briefly, anti-CD3 and anti-PD-L1 antibodies on the nanosystem enable simultaneous crosslinking of CD3 on the T cell surface and PD-L1 on the tumor surface, which promote the formation of synapses [32,33]. FeC-G@M-C&P maintains the stability of synapses, transmits T cell activation signals, and secretes cytokines and perforin, resulting in T cell-mediated cytotoxicity. However, only anti-PD-L1 antibody-modified FeC-G@M-P fails to form synapses and triggers T cell activation. Compared with the existing CD3-targeted bispecific antibodies, FeC-G@M-C&P possesses the following advantages and drawbacks: FeC-G@M-C&P can simultaneously crosslink multiple antibody molecules, improving the efficiency of synapse formation and the T cell-mediated cytotoxicity against C1498 cells. In addition, FeC-G@M-C&P can achieve synergistic effects, including CDT, CXCR4/CXCL12 blockade therapy, T cell recruitment and PD-L1 blockade, which cannot be achieved by pure protein bispecific antibodies. However, FeC-G@M-C&P faces challenges, such as multistep coupling procedure, that make quality control difficult.

Activated T cells secret cytokines including IFN-γ, interleukin, perforin and granzyme B to the lysis tumor cell. Therefore, we measured the related levels of cytokines. As shown in Figure 5C–E, the secretion of TNF-α, IL-2 and IFN-γ markedly elevated in the FeC-G@M-C&P + T cells group relative to the T cells group. Moreover, the levels of perforin and granzyme B in the FeC-G@M-C&P + T cells group was also significantly higher than that in the T cells group, indicating that FeC-G@M-C&P could effectively trigger T cell activation (Figure S7). Taken together, FeC-G@M-C&P could effectively activate T cells and promote the secretion of antitumor cytokines, thus ultimately enhancing T cell-mediated cytotoxicity.

Then, the anti-leukemia effect of FeC-G@M-C&P was evaluated by CCK-8 assay. As shown in Figure 5F, cell viability of Glucose + FeC-G, Glucose + FeC-G@M and Glucose + FeC-G@M-P groups decreased compared with the control group, suggesting the efficiency of CDT. Cell viability of Glucose + FeC-G@M-P was lower than that of the Glucose + FeC-G@M and Glucose + FeC-G group due to the improvement of ROS production and alleviation of PD-L1-mediated immune suppression. The Glucose + FeC-G@M-C&P + T cells group showed the lowest cell viability (19.1 ± 6.3%) among all the groups, suggesting the synergistic anti-leukemia effects by integrating CDT, PD-L1 immunosuppression reversal and T cells recruitment.

### 3.6. Evaluation of Biosafety and Biocompatibility

**Figure 6:**
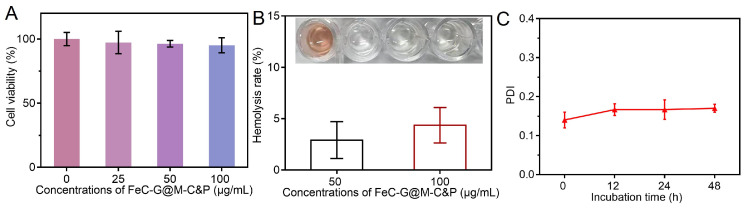
(**A**) Viability of HUVECs treated with different concentrations of FeC-G@M-C&P for 48 h (*n* = 3). (**B**) Hemolysis rates of RBCs treated with different concentrations of FeC-G@M-C&P for 30 min (*n* = 3). The photographs from left to right represent the pure water, PBS, 50 μg/mL FeC-G@M-C&P, and 100 μg/mL FeC-G@M-C&P groups. (**C**) PDI of FeC-G@M-C&P incubated in PBS buffer at different time points (*n* = 3).

Biosafety and biocompatibility assessments are necessary for biomedical application. We tested cytotoxicity on normal cells, hemolysis rate, and stability under physiological conditions for assessing biosafety and biocompatibility of nanosystem. CCK-8 assay was performed to detect viability of HUVECs treated with different concentrations of FeC-G@M-C&P. The results demonstrated that FeC-G@M-C&P exhibited negligible cytotoxicity against normal cells under treatment concentrations (Figure 6A). Next, hemolysis assay was conducted to quantify the hemolytic degree. As shown in Figure 6B, hemolysis rates were below 5%, verifying the outstanding blood compatibility of FeC-G@M-C&P. PDI is a parameter evaluating the size uniformity of the nanosystem. No remarkable rise in PDI is observed during incubation, supporting the robust colloidal stability of the nanosystem. Then, we detected PDI values at different time points using dynamic light scattering. As exhibited in Figure 6C, the PDI values remained with no significant change within 48 h incubation, indicating the good physiological stability of FeC-G@M-C&P. Collectively, FeC-G@M-C&P possessed excellent biosafety and biocompatibility.

Analysis of the degradation profiles and clearance routes of the nanosystem shows great significance to fundamental research and clinical translation. Next, we discuss the degradation and clearance routes of FeC-G@M-C&P for revealing its biological fate and improving the biosafety evaluation system. The FeC-G@M-C&P exhibits a sequential degradation pattern: the antibodies and cell membrane are digested by related enzymes in plasma and lysosomes, followed by hydrolysis of imine bonds in lysosomes after cellular internalization, which release iron porphyrin and iron ions. Meanwhile, ROS catalyzed by iron porphyrin further oxidize aromatic skeleton and accelerate degradation. In terms of clearance routes, the nanosystems (>6 nm) are predominantly captured by Kupffer cells of the reticuloendothelial system, and the medium-sized degraded fragment debris is excreted via the biliary-fecal pathway [34]. Iron ions and tiny degraded fragments undergo renal excretion. Although cellular experiments confirm the preliminary biosafety of FeC-G@M-C&P, its systemic safety remains challenging, such as the degradation of FeC-G@M-C&P within Kupffer cells induced iron overload, chronic oxidative injury and potential inflammatory risks. In further work, comprehensive toxicology tests are vital to perfect the biosafety evaluation for subsequent biomedical transformation.

## 4. Conclusions

In this work, we constructed a bispecific nanosystem (FeC-G@M-C&P) for combined AML therapy. The prepared FeC-G@M-C&P possessed good cascade catalytic activity. Experiments including targeting capability detection, ROS generation ability evaluation and cell apoptosis analysis confirmed that a modification nanosystem with bone marrow stromal cell membrane could enhance targeting ability, accelerate ROS production and realize CDT. Furthermore, the cell membrane could prevent the migration and adhesion of C1498 cells via interfering CXCR4/CXCL12 axis. The constructed FeC-G@M-C&P could integrate CDT, CXCR4/CXCL12 axis blockade therapy and immunotherapy, which provides a favorable combined treatment strategy to achieve improved anti-leukemia effects.

## Data Availability

The original contributions presented in this study are included in the article/Supplementary Material. Further inquiries can be directed to the corresponding authors.

## Supplementary Materials

The following supporting information can be downloaded at https://www.mdpi.com/article/10.3390/ma19143001/s1: Figure S1: XPS spectra of (A) Fe 2p, (B) O 1s, (C) N 1s, (D) C 1s, (E) Cl 2p, and (F) Br 3d; Figure S2: PDI of FeC and FeC-G; Figure S3: Absorption spectra of FeC and FeC-G evaluated by a UV–vis spectrophotometer; Figure S4: Zeta potential distribution of (A) FeC and (B) FeC-G; Figure S5: Fluorescence spectroscopy of nanosystems with different formulation (Excitation wavelength: 650 nm); Figure S6: Viability of C1498 cells after incubation with T cells, FeC-G@M-P + T cells, and FeC-G@M-C&P + T cells for 24 h (*n* = 3); Figure S7: Relative levels of (A) perforin and (B) granzyme B in cell culture supernatants in different treatment groups (*n* = 3); Table S1: The components of abbreviations.

## Abbreviations

The following abbreviations are used in this manuscript:

·OH	Hydroxyl radicals
H_2_O_2_	Hydrogen peroxide
POD	Peroxidase
GOX	Glucose oxidase
ROS	Reactive oxygen species
TPyP	5,10,15,20-tetra(4-pyridyl)-21H,23H-porphine
TMB	3,3′,5,5′-tetramethylbenzidine
ABTS	2,2′-azino-bis-(3-ethylbenzthiazoline-6-sulphonate)
MeOH	Methanol
MP	1-methyl-2-pyrrolidinone
XD	p-xylylene dibromide
PVP	Polyvinylpyrrolidone
FeCl_2_·4H_2_O	Ferrous chloride tetrahydrate
DMEM	Dulbecco’s modified eagle medium
FBS	Fetal bovine serum
CCK-8	Cell counting kit-8
ELISA	Enzyme-linked immunosorbent assay
TEM	Transmission electron microscopy
XPS	X-ray photoelectron spectroscopy
TGA	Thermal gravimetric analysis
EDS	Energy dispersive spectroscopic
DCFH-DA	2′,7′-dichlorodihydrofluorescein diacetate
TNF-α	Tumor necrosis factor-alpha
IL-2	Interleukin-2
IFN-γ	Interferon-gamma
PDI	Polymer dispersity index
