# Supplementary material for: Covalent Organic Framework Bispecific Nanosystem for the Combined Treatment of Acute Myeloid Leukemia
Source: Materials (Basel). 2026 Jul 12;19(14):3001. doi: 10.3390/ma19143001 (PMC13413223; doi:10.3390/ma19143001)
Supplement: Supplementary file 1 [file materials-19-03001-s001.zip › materials-4313762-supplementary.pdf]

Covalent Organic Framework Bispecific Nanosystem for the Combined Treatment of Acute Myeloid Leukemia

Huiyuan Bai [1,*], Mengsi Lin [2], Yiming Xia [2], Xi Gu [1], Maorong Jiang [1] and Dengbing Yao [1,2,*]

1 School of Life Sciences, Key Laboratory of Neuroregeneration of Jiangsu and Ministry of Education, Co-Innovation Center of Neuroregeneration, Nantong University, Nantong 226019, China;
ntdxlotus@163.com (X.G.); jiangmr@ntu.edu.cn (M.J.)

2 Medical School of Nantong University, Nantong University, Qixiu Campus, Nantong 226001, China;
wsdmn18@126.com (M.L.); 18822355805@163.com (Y.X.)

* Correspondence: hybai@ntu.edu.cn (H.B.); yaodb@ntu.edu.cn (D.Y.)

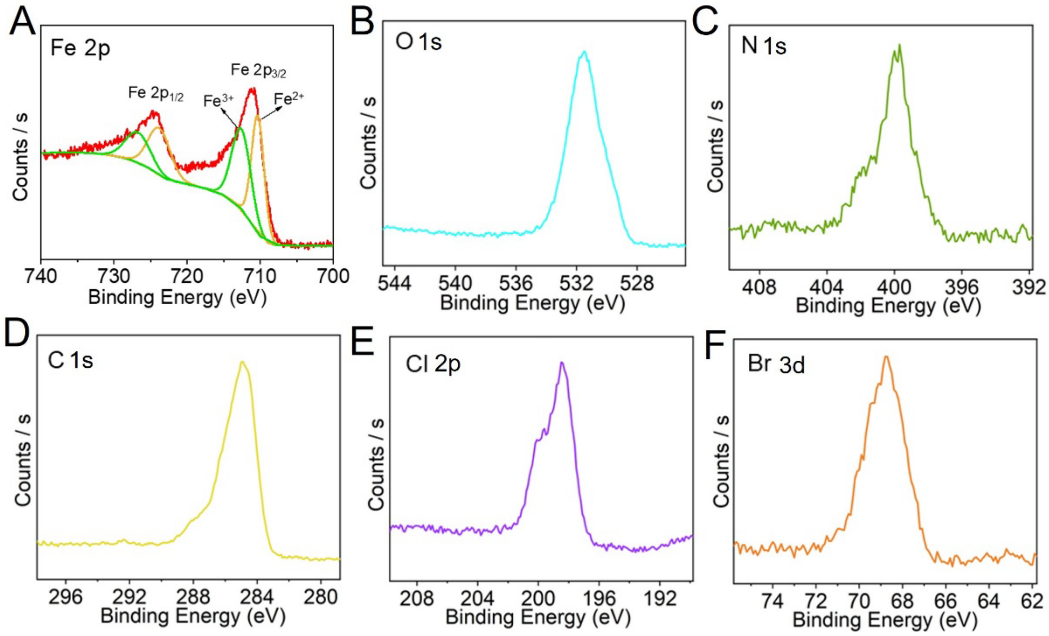

Figure S1. XPS spectra of (A) Fe 2p, (B) O 1s, (C) N 1s, (D) C 1s, (E) Cl 2p, and (F) Br 3d.

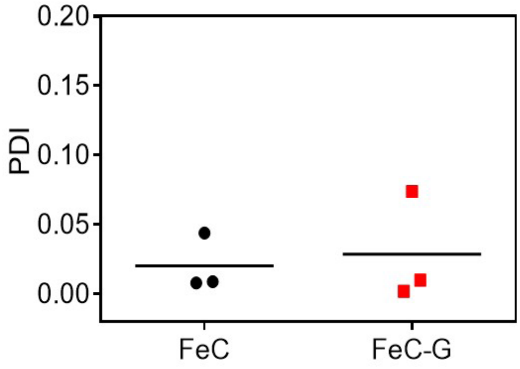

Figure S2. PDI of FeC and FeC-G.

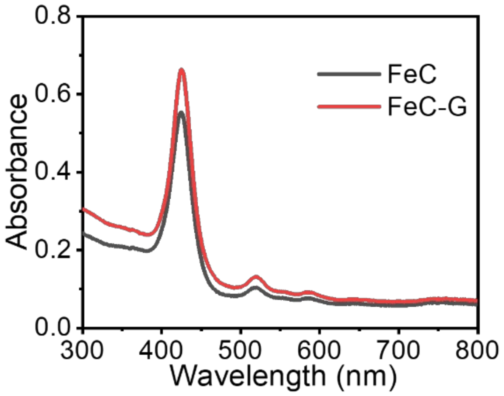

Figure S3. Absorption spectra of FeC and FeC-G evaluated by a UV–vis spectrophotometer.

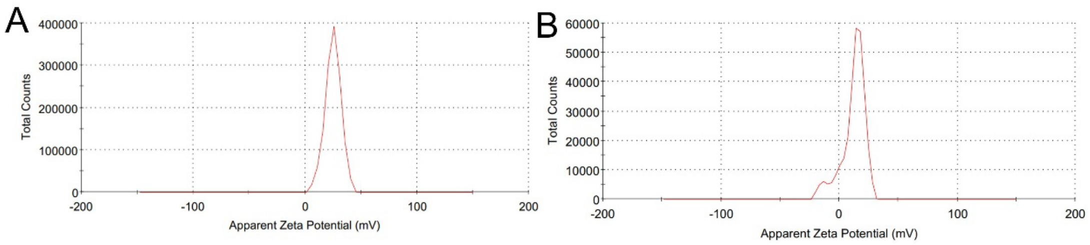

Figure S4. Zeta potential distribution of (A) FeC and (B) FeC-G.

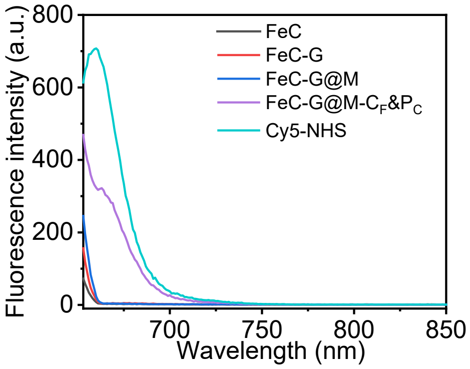

Figure S5. Fluorescence spectroscopy of nanosystems with different formulation (Excitation wavelength: 650 nm).

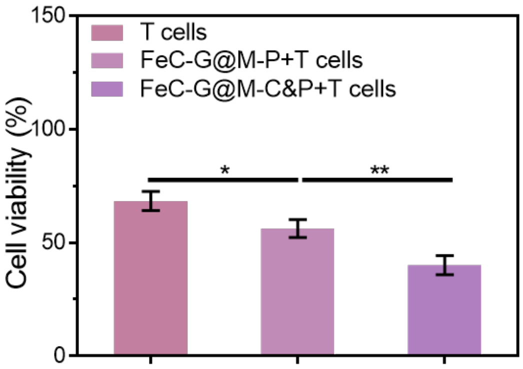

Figure S6. Viability of C1498 cells after incubation with T cells, FeC-G@M-P+T cells, and FeC-G@M-C&P+T cells for 24 h (n = 3).

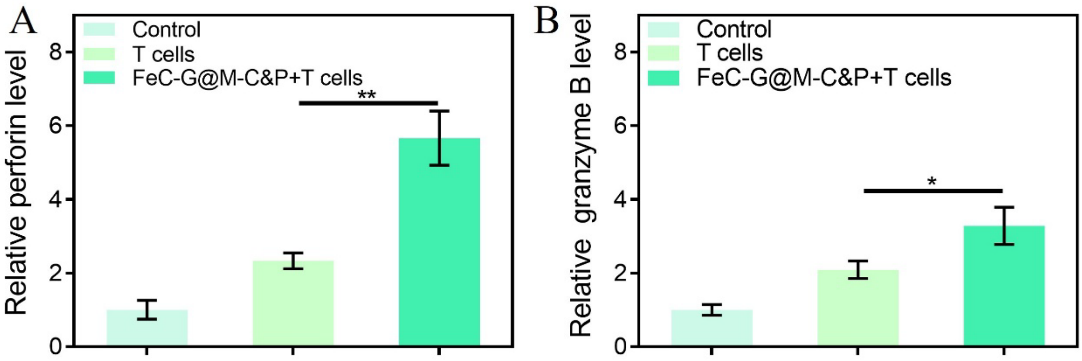

Figure S7. Relative levels of (A) perforin and (B) granzyme B in cell culture supernatants in different treatment groups (n = 3).

Table 1. The components of abbreviations

Abbreviations	Components
FeC	Iron porphyrin-based covalent organic framework
FeC@M	Iron porphyrin-based covalent organic framework, bone marrow stromal cell membrane
FeC@M-P	Iron porphyrin-based covalent organic framework, bone marrow stromal cell membrane, anti-PD-L1 antibody
FeC-G	Iron porphyrin-based covalent organic framework, glucose oxidase
FeC-G@M	Iron porphyrin-based covalent organic framework, glucose oxidase, bone marrow stromal cell membrane
FeC-G@M-P	Iron porphyrin-based covalent organic framework, glucose oxidase, bone marrow stromal cell membrane, anti-PD-L1 antibody
FeC-G@M-C&P	Iron porphyrin-based covalent organic framework, glucose oxidase, bone marrow stromal cell membrane, anti-CD3 antibody, anti-PD-L1 antibody
FeC-G@M-C_F&P_C	Iron porphyrin-based covalent organic framework, glucose oxidase, bone marrow stromal cell membrane, FITC-labeled anti-CD3 antibody, Cy5-labeled anti-PD-L1 antibody
